# Serially measured pre-diagnostic levels of serum cytokines and risk of brain cancer in active component military personnel

**DOI:** 10.1038/s41416-018-0272-x

**Published:** 2018-10-09

**Authors:** Alina V. Brenner, Peter D. Inskip, Jennifer Rusiecki, Charles S. Rabkin, Joshua Engels, Ruth M. Pfeiffer

**Affiliations:** 1 Radiation Effects Research Foundation, Hiroshima, Japan; 2 Division of Cancer Epidemiology and Genetics, National Cancer Institute, NIH, Rockville, Maryland USA; 3 Department of Preventive Medicine and Biostatistics, F. Edward Hébert School of Medicine, Uniformed Services University of the Health Sciences, Bethesda, MD USA

**Keywords:** Risk factors, Interleukins

## Abstract

**Background:**

There is growing evidence that history of allergic or autoimmune disease is associated with reduced risk of glioma, but few prospective studies have explored the biological basis. To assess associations with immune conditions and levels of 14 cytokines in serial prediagnostic serum samples, we conducted a study of glioma/brain cancer nested in a cohort of active component military personnel.

**Methods:**

A total of 457 case-control sets were ascertained from the Department of Defense (DoD) Automated Central Tumour Registry, Defense Medical Surveillance System (DMSS) database, and DoD Serum Repository. These were individually matched on sex, race/ethnicity, birth year, number of serum samples (1, 2 or 3), and date(s) of sample collection. We obtained diagnoses of pre-existing immune-related conditions from the DMSS database and measured cytokines using Meso Scale Discovery assays. Statistical analyses included conditional logistic regression.

**Results:**

Overall association between glioma and prior immune-related conditions was null. Higher levels of IL-15 and IL-16 were independently associated with lower glioma risks (*P*_trend_ = 0.002 and *P*_trend_ = 0.001); both associations were more pronounced in individuals with prior immune conditions (*P*_heterogeneity_ = 0.0009 and *P*_heterogeneity_ = 0.031).

**Conclusions:**

Associations with pre-diagnostic levels of IL-15 and IL-16 and their modification by diagnosis of immune-related conditions support the importance of immune alterations in glioma aetiology years before diagnosis.

## Introduction

There is accumulating evidence that prior history of allergy and, to a lesser extent, autoimmune disease is associated with reduced risk of glioma,^1–3^ the most common type of primary brain cancer in adults.^4^ These associations are reported in multiple case-control studies^1,2^ and several prospective studies.^5,6^ Some studies have also reported associations of common single nucleotide polymorphisms (SNPs) in cytokine genes (*IL4*, *IL4RA*, *IL13*, *IL6, SELP*) with risk of glioma development^7–12^ and/or survival.^13^ It is notable that variants in the same genes are also associated with risk of allergic and/or autoimmune diseases,^14,15^ although the mechanistic links between these observations are unclear. While allergies and autoimmune diseases differ in underlying immune alterations, both result from an inappropriate immune response to otherwise innocuous foreign substances. How chronic antigenic stimulation accompanying allergic or autoimmune diseases may be protective against glioma remains largely unknown, but changes in cellular/humoral immunity and levels of associated cytokines may be involved.^16^ In addition, there is intriguing recent evidence that selected growth factors, a subset of cytokines governing organogenesis, are also altered in allergy and autoimmune disease and may drive glioma stem cell proliferation and invasion; the latter has been shown in mouse models and in vitro studies,^17^ but human data are limited.

Due to the rarity of glioma, case-control studies generally have been the preferred study design and have provided initial clues about the role of immune-related conditions in the aetiology of glioma; however, most case-control studies of glioma in relation to immune markers suffer from the limitations of having to rely on serum samples collected after cancer diagnosis, and possibly after initiation of treatment.^10,13,18,19^ Furthermore, due to the aggressive and rapidly progressive nature of adult gliomas, patients may die or become incapacitated before they provide their medical history. Studies with serum samples and validated information on medical history collected prior to glioma diagnosis are less prone to bias but are limited. Several case-control studies nested in prospective cohorts reported an inverse association between elevated levels of total IgE in pre-diagnostic serum samples and glioma risk.^20–22^ Also, a recent case-control study nested in the Janus Serum Bank Cohort (JSBC) in Norway assessed 277 cytokines and identified five (sIL10RB, VEGF, beta-Catenin, CCL22, and LIF) independently related to glioma risk in one of five periods prior to glioma diagnosis based on a single pre-diagnostic sample.^23,24^ These findings remain unconfirmed.

Using the Department of Defense (DoD) Automated Central Tumour Registry (ACTUR),^25^ Defense Medical Surveillance System (DMSS) database and DoD Serum Repository (DoDSR),^26,27^ we conducted a nested case-control study to assess associations of risk of adult glioma/brain cancer with pre-diagnostic serum levels of 14 a priori selected cytokines and growth factors and a personal history of immune-related conditions. At the time of selection and measurements of these cytokines, the JSBC study results were unknown. We used multiple, prospectively collected serum samples and accurate medical diagnoses for active component U.S. military personnel. The availability of serial serum samples allowed us to evaluate whether cytokine associations vary by time leading up to the diagnosis of glioma/brain cancer.

## Materials and Methods

### Study population

The current case-control study of brain cancer is nested within a population of more than 10 million U.S. military applicants, active component personnel, and reserve service members who had serum samples collected since 1988 and stored in the DoDSR.^26,27^ These samples are collected as part of a universal, mandatory screening for human immunodeficiency virus (HIV-1) implemented by the DOD. The first archived serum sample of each service member is typically obtained during a pre-induction medical examination or shortly after military service commences. Follow-up samples generally consist of serum that remains after routine, periodic HIV-1 antibody testing or required pre- and post-deployment samples, carried out on average every two years for a given individual. At the time of the study, the DoDSR policy allowed use of up to three serum samples per individual for research purposes.

We selected eligible cases from active component military personnel with a diagnosis of a first primary brain glioma [International Classification of Diseases for Oncology, 3rd edition (ICD-O-3) codes 938-948] between 1988 and 2006 in the ACTUR database (*N* = 162 cases; median age at diagnosis, 29 years), or a first primary brain cancer [International Classification of Diseases, 9th revision (ICD-9) code 191] supported by relevant diagnostic procedure codes [Current Procedural Terminology (CPT) codes for nervous system surgery: 61000-64999, cytopathology: 88104-88199, cytogenetics: 88230-88299 or surgical pathology: 88300-88399] in the DMSS database (*N* = 295 cases; median age at diagnosis, 30 years) and at least one serum sample in the DoDSR collected at least 12 months prior to diagnosis of glioma or brain cancer. During selection, we made certain that no duplicate cases from both ACTUR and DMSS were included in the study. Ninty four percent of cases identified through ACTUR had ICD-O-3 codes consistent with astrocytoma or other glioma. Of these, low-grade astrocytoma, oligodendroglioma, and mixed oligoastrocytoma accounted for 80%. We lack tumour morphology codes for the primary brain cancer cases ascertained through DMSS but, based on comparability of median ages at diagnosis for ACTUR and DMSS cases and age-specific and tumour histology-specific distribution of cases in ACTUR and Central Brain Tumour Registry of the United States (CBTRUS) databases,^4^ we presume that most DMSS cases were low-grade glioma. For purposes of the present analysis, the term “brain cancer” is nearly interchangeable with “glioma”.

Eligible controls were active component military personnel free of brain cancer or other neoplasm in the DMSS database (ICD-9 codes outside the range 140–239) with at least one serum sample in the DoDSR database. Controls were randomly selected from the pool of eligible subjects and individually matched to cases in a 1:1 ratio on sex, year of birth (+/− 1 year), race/ethnicity (white, black, other), number of serum samples (1, 2 or 3), and date of each serum sample collection (+/− 30 days). We defined a reference date for the case and matched control as the date of brain cancer diagnosis. For individuals with more than three serum samples collected ≥12 months before the reference date, we requested the earliest and latest samples and an intermediate sample spaced evenly between the earliest and latest samples wherever possible.

The study protocol was reviewed and approved by the Institutional Review Boards of the Uniformed Services University and Armed Forces Institute of Pathology, and the Office of Human Subjects Research Protections of the National Cancer Institute.

### Medical history data

After the case-control sets were identified, data on diagnoses of immune-related conditions and diseases were extracted from in-patient and out-patient medical records stored in the DMSS database. A complete list of requested ICD-9 codes is presented in Supplemental Table S1. The date of immune condition diagnosis was taken as the earliest date the condition of interest appeared in the DMSS database. In the main analyses, we considered conditions that were diagnosed ≥12 months prior to the reference date and grouped them as follows: (1) any allergy, including hay fever, asthma, eczema, or other allergy; (2) any autoimmune disease, including organ-specific and systemic diseases; and (3) either allergy or autoimmune disease. We also conducted sensitivity analyses considering only conditions diagnosed ≥24 months prior to the reference date as in Cahoon et al.^6^

### Laboratory assays

All serum samples (0.5 ml) were continuously frozen at approximately –30 °C until cytokine testing. In total, we measured 14 *a* priori selected cytokines and growth factors in 2305 unique samples. The assays were conducted at the SAIC-Frederick laboratory using a combination of four sensitive custom V-PLEX Meso Scale Discovery kits (MSD, Gaithersburg, MD) including human Proinflammatory Panel 1 (IFNγ, IL-8, IL-10, TNFα), Cytokine Panel 1 (IL-7, IL12P40, IL-15, IL-16), Chemokine Panel 1 (TARC, MCP1), and Angiogenesis Panel 1 (VEGF, PLGF). We also used standard MSD kits to measure human HGF and TGFβ individually. MSD platforms use an electro-chemiluminescence detection system that is designed to minimise nonspecific reactions and background noise. All assays were performed according to manufacturer instructions.

To measure study samples including quality control (QC) samples, 33 × 96-well plates were necessary. Case-control sets were selected for plating at random. All serum samples for a given set were plated on the same plate in random order in adjacent wells. QC samples included three external pooled serum samples that were measured in duplicate on all plates (3 × 2 × 33 = 198 measurements) and 110 internal study samples of which 55 were measured in duplicate on the same plate (110 measurements) and 55 were measured on different plates (110 measurements). The coefficients of variation ranged from 5.4 to 14.3 and the intraclass correlation coefficients ranged from 95% to 99%. Fourteen percent of the IL-8 measurements and 15% of the IL-10 measurements were below detection limits, while, for the remaining 12 cytokines, <2% of values were below detection limits.

### Statistical analysis

Conditional logistic regression models were used to estimate hazard ratios (HR) and calculate 95% confidence intervals (95% CI) for associations between brain cancer and concentration of each cytokine, as well as history of immune-related conditions. We conditioned on the matched sets, and also on sample number, and thus compared levels of the first, second or third cytokine measurement in a case with those in the matched control. To account for correlations among multiple measurements from the same matched set, we computed robust sandwich variance estimates. The conditioning was used to accommodate the matched design. Cytokine measurements were analysed as categorical (quartiles, based on distribution in controls, see Table S2), ordinal (ordered quartiles), and, where indicated, continuous log-transformed variables. All analyses were additionally adjusted for type of military service (Army, Navy, Air Force, Marine, and Coast Guard), because branch of service was found to be associated with brain cancer risk in preliminary analyses. We analysed effects of immune-related conditions and cytokine levels both separately and simultaneously in the same model to evaluate mutual confounding.

We also explored effect modification of the associations with cytokine levels by diagnosis of immune-related condition, lag-time to brain cancer diagnosis (or reference date for controls), and age at brain cancer diagnosis (reference date). The effect modifiers were evaluated individually and then simultaneously, i.e., mutually adjusting for each other. We also conducted separate analyses for brain cancer cases ascertained through ACTUR and DMSS databases.

All tests of statistical significance were two-sided with type-I error of 0.05. Analyses were carried out in SAS version 9.3.^28^

## Results

Descriptive characteristics of the 457 cases (overall and according to source of case ascertainment) and 457 controls are presented in Table 1. As controls were individually matched to cases on sex, race, year of birth, age, number of serum samples and time between blood collection and reference date, these variables had nearly identical distributions. Almost 89% of all cases and controls were males, 81% were white; 81% were born between 1945 and 1983 (median year, 1973); and 83% were between 18 and 39 years old at diagnosis/reference date (median age, 30 years). There was a higher proportion of Air Force and Coast Guard personnel and a lower proportion of Marines and Navy among all cases compared with controls (*P* < 0.001). ACTUR and DMSS cases were comparable with respect to many sociodemographic characteristics except year of birth and type of military service. Specifically, DMSS cases tended to be born in later calendar years than ACTUR cases and had a lower proportion of Army personnel. Sixty-seven percent of all cases and controls had three pre-diagnostic serum samples, 18% had two, and 15% had one serum sample. The proportion of cases with three serum samples was higher in DMSS than ACTUR cases (75% vs. 53%, respectively). Overall, nearly 50% of samples were taken at least 3.6 years prior to cancer diagnosis (up to 22 years); with the respective proportions in DMSS and ACTUR cases being 49% and 55%, respectively.

**Table 1:** Selected descriptive characteristics of cases (overall and according to source of case ascertainment) and controls in the study of brain cancer among active component military personnel

Characteristic	Controls *N* = 457 (%)	Cases *N* = 457 (%)	ACTUR *N* = 162 (%)	DMSS *N* = 295 (%)
Sex^a^				
Male	406 (88.8)	406 (88.8)	142 (87.7)	264 (89.5)
Female	51 (11.1)	51 (11.1)	20 (12.4)	31 (10.5)
Race^a^				
White	371 (81.2)	371 (81.2)	130 (80.3)	241 (81.7)
Black	52 (11.4)	52 (11.4)	23 (14.2)	29 (9.8)
Other	16 (3.5)	16 (3.5)	2 (1.2)	14 (4.8)
Unknown	18 (3.9)	18 (3.9)	7 (4.3)	11 (3.7)
Reference age (years)^a,b^				
18–20	37 (8.1)	38 (8.3)	16 (9.9)	22 (7.5)
21–24	94 (20.6)	95 (20.8)	34 (21.0)	61 (20.7)
25–29	99 (21.7)	99 (21.7)	36 (22.2)	63 (21.4)
30–34	69 (15.1)	69 (15.1)	24 (14.8)	45 (15.3)
35–39	82 (17.9)	80 (17.5)	31 (19.1)	49 (16.6)
40–61	76 (16.6)	76 (16.6)	21 (13.0)	55 (18.6)
Year of birth^a^				
1945–1963	81 (17.7)	81 (17.7)	45 (27.8)	36 (12.2)
1964–1969	79 (17.3)	78 (17.1)	43 (26.5)	35 (11.9)
1970–1974	105 (23.0)	99 (21.7)	36 (22.2)	63 (21.4)
1975–1983	106 (23.2)	111 (24.3)	29 (17.9)	82 (27.8)
1984–1991	86 (18.8)	88 (19.3)	9 (5.6)	79 (26.8)
Military service^b,c^				
Army	163 (35.7)	164 (35.9)	67 (41.4)	97 (32.9)
Navy	156 (34.1)	118 (25.8)	44 (27.2)	74 (25.1)
Air force	63 (13.8)	104 (22.8)	31 (19.1)	73 (24.8)
Marines	74 (16.2)	61 (13.4)	19 (11.7)	42 (14.2)
Coast guard	1 (0.2)	10 (2.2)	1 (0.6)	9 (3.1)
Number of pre-diagnostic serum samples^a^				
One	71 (15.5)	70 (15.3)	41 (25.3)	29 (9.8)
Two	80 (17.5)	81 (17.7)	36 (22.2)	45 (15.3)
Three	306 (67.0)	306 (67.0)	85 (52.5)	221 (74.9)
Time between serum collection and reference year (years)^c,d^				
1.0–1.8	287 (25.0)	286 (24.9)	56 (15.2)	230 (29.4)
1.9–3.5	280 (24.4)	282 (24.5)	110 (29.9)	172 (22.0)
3.6–7.1	282 (24.5)	286 (24.9)	97 (26.4)	189 (24.2)
7.2–22.1	300 (26.1)	296 (25.7)	105 (28.5)	191 (24.4)

ACTUR automated central tumour registry, DMSS defense medical surveillance system

^a^Matching variables

^b^Age at diagnosis of brain cancer/glioma for cases and respective date for controls

^c^*P* value for test of heterogeneity of proportions in all cases and controls <0.001

^d^For all serum samples

We found no evidence that a history of individual or grouped immune-related conditions diagnosed ≥12 months (Table 2) or ≥24 months (Table S3) prior to the reference date among individuals with available serum was associated with overall risk of brain cancer. These associations did not vary by proximity of the diagnosis dates (≤median interval vs. >median interval) between the immune-related condition and brain cancer or age at brain cancer diagnosis (<35 vs. ≥35 years) (data not shown).

**Table 2:** Association between immune-related conditions diagnosed ≥12 months prior to the reference date and risk of brain cancer among active component military personnel

Immune-related condition	Controls *N* = 457 (%)	Cases *N* = 457 (%)	HR	95% CI
Any allergy	113 (24.7)	109 (23.9)	0.86	0.60, 1.24
Hay fever	50 (10.9)	59 (12.9)	1.06	0.68, 1.66
Asthma	10 (2.2)	13 (2.8)	1.38	0.57, 3.31
Eczema	55 (12.0)	56 (12.3)	0.98	0.65, 1.46
Other	22 (4.8)	22 (4.8)	1.03	0.54, 1.95
Any autoimmune disease	14 (3.1)	16 (3.5)	1.01	0.48, 2.12
Organ-specific	12 (2.6)	16 (3.5)	1.23	0.57, 2.66
Systemic	3 (0.7)	0	NE^a^	
Any allergy or autoimmune disease	117 (25.6)	117 (25.6)	0.91	0.64, 1.30

*HR* hazard ratio, 95% *CI* 95% confidence interval, *NE* not estimable

^a^*P* value for Fisher’s exact test = 0.12

The intra-class correlation coefficients (ICCs) for cytokine concentrations in controls with repeated samples varied widely (Table S4). The ICCs were reasonably good (>60%) for IL-12p40 and IL-15, fair (≥40% and <59%) for IL-7, TARC, PLGF, VEGF and HGF, and poor (<40%) for IL-16, MCP1, IFNg, IL-10, IL-8, TNFa and TGFβ.

Of 14 cytokines examined, significant inverse associations with brain cancer risk were found for IL-15 and IL-16 when these were modelled either as a quartile trend (*P* = 0.002 and *P* = 0.001, respectively) or a log-linear trend (*P* = 0.001 and *P* = 0.004, respectively) (Table 3). The associations with IL-10 and TGFβ reached significance only based on one test: quartile trend for IL-10 (*P* = 0.033) and log-linear trend for TGFβ (*P* = 0.049). When included in the same model, the associations with IL-15 and IL-16 persisted, while the associations with IL-10 and TGFβ became attenuated and were no longer significant (data not shown). There was no indication that the associations with cytokine levels differed meaningfully between ACTUR and DMSS cases (Table S5). When analyses were limited to serum samples collected ≥24 months prior to the reference date, the results did not change materially (Table S6).

**Table 3:** Associations between pre-diagnostic levels of serum cytokines and risk of brain cancer among active component military personnel

Cytokine		Quartiles of cytokine concentration					
		Q1^a^	Q2	Q3	Q4	*P* ^b^	*P* ^c^
IL-12p40	HR^d^	1.00	1.06	1.12	1.19	0.333	0.285
	95% CI		0.79, 1.42	0.81, 1.57	0.83, 1.70		
IL-15	HR	1.00	0.83	0.73	0.55	0.002	0.001
	95% CI		0.63, 1.09	0.53, 1.00	0.38, 0.80		
IL-16	HR	1.00	0.89	0.71	0.62	0.001	0.004
	95% CI		0.68, 1.15	0.53, 0.94	0.46, 0.84		
IL-7	HR	1.00	0.95	1.18	1.18	0.155	0.198
	95% CI		0.72, 1.26	0.89, 1.58	0.86, 1.62		
MCP1	HR	1.00	0.87	0.90	0.79	0.231	0.148
	95% CI		0.66, 1.15	0.65, 1.22	0.56, 1.11		
TARC	HR	1.00	1.25	1.04	1.09	0.883	0.421
	95% CI		0.93, 1.68	0.76, 1.41	0.77, 1.54		
PLGF	HR	1.00	0.95	0.96	1.04	0.844	0.698
	95% CI		0.71, 1.26	0.70, 1.32	0.72, 1.50		
VEGF	HR	1.00	1.25	1.42	1.14	0.417	0.317
	95% CI		0.92, 1.70	1.02, 1.98	0.80, 1.62		
IFNg	HR	1.00	1.00	1.06	1.08	0.771	0.120
	95% CI		0.78, 1.29	0.81, 1.39	0.76, 1.40		
IL-10	HR	1.00	1.03	0.87	0.73	0.033	0.067
	95% CI		0.78, 1.37	0.64, 1.17	0.52, 1.01		
IL-8	HR	1.00	1.18	0.95	0.98	0.559	0.727
	95% CI		0.88, 1.58	0.69, 1.30	0.71, 1.35		
TNFa	HR	1.00	1.08	1.10	1.09	0.656	0.291
	95% CI		0.81, 1.43	0.81, 1.50	0.78, 1.50		
HGF	HR	1.00	0.82	0.87	0.80	0.228	0.584
	95% CI		0.63, 1.06	0.66, 1.15	0.58, 1.10		
TGFb1	HR	1.00	1.34	1.35	1.38	0.080	0.049
	95% CI		1.03, 1.74	1.01, 1.82	0.99, 1.92		

*Q1, Q2, Q3, Q4* quartiles of cytokine concentration in controls, see absolute values in Supplemental Table S2, *HR* hazard ratio, *95% CI* 95% confidence interval

^a^Referent quartile

^b^*P* value for continuous quartile trend in cytokine concentration

^c^*P* value for continuous trend in log-transformed cytokine concentration

^d^All hazard ratios are adjusted for type of military service and represent change in risk per quartile increase in cytokine concentration

The associations with IL-15, IL-16 and PLGF based on a quartile trend varied significantly by history of immune-related conditions (*P* = 0.0009, *P* = 0.031, *P* = 0.045, respectively; Table 4) and were more pronounced in individuals with a positive than negative history. The evidence for a cytokine trend in individuals with and without immune-related conditions based on a log-linear model was largely consistent with that based on a quartile trend (Table S7).

**Table 4:** Associations between pre-diagnostic levels of serum cytokines and risk of adult brain cancer among active component military personnel overall and according to diagnosis of immune-related condition

Cytokine	Overall		Immune-related condition				*P* ^d^
			No		Yes		
	Case-control	HR^a^	Case-control	HR	Case-control	HR	
	N^b^	95% CI	N^c^	95% CI	N^c^	95% CI	
IL-12p40	455	1.06	242	1.08	59	1.70	0.475
		1.00, 1.12		0.93, 1.25		1.13, 2.54	
IL-15	455	0.83	242	0.87	59	0.64	0.0009
		0.74, 0.94		0.75, 1.02		0.42, 0.99	
IL-16	450	0.85	239	0.85	58	0.70	0.031
		0.77, 0.94		0.75, 0.96		0.54, 0.90	
IL-7	454	1.08	241	1.09	59	1.42	0.310
		0.97, 1.19		0.96, 1.25		1.03, 1.96	
MCP1	453	0.94	240	0.94	59	0.70	0.063
		0.84, 1.04		0.82, 1.08		0.49, 0.99	
TARC	448	1.01	237	1.03	58	0.87	0.809
		0.90, 1.13		0.89, 1.19		0.60, 1.28	
PLGF	454	1.01	243	1.08	59	0.62	0.045
		0.90, 1.14		0.93, 1.26		0.42, 0.89	
VEGF	452	1.05	242	1.09	59	0.91	0.636
		0.94, 1.18		0.94, 1.27		0.66, 1.26	
IFNg	451	1.01	241	0.99	57	1.19	0.395
		0.92, 1.12		0.87, 1.12		0.87, 1.62	
IL-10	381	0.89	176	0.88	45	0.91	0.152
		0.80, 0.99		0.77, 1.01		0.66, 1.24	
IL-8	378	0.97	194	0.92	50	0.74	0.880
		0.88, 1.08		0.81, 1.06		0.53, 1.02	
TNFa	434	1.02	228	1.01	57	0.85	0.827
		0.92, 1.14		0.87, 1.17		0.66, 1.10	
HGF	454	0.94	243	0.97	59	0.83	0.178
		0.85, 1.04		0.85, 1.11		0.61, 1.13	
TGFb1	452	1.10	242	1.15	59	0.96	0.155
		0.99, 1.23		0.99, 1.32		0.68, 1.34	

*HR* hazard ratio, *95% CI* 95% confidence interval

^a^All hazard ratios are adjusted for type of military service and represent change in risk per quartile increase in cytokine concentration

^b^Total number of case-control pairs included in the analysis

^c^Number of informative case-control pairs included in the analysis

^d^All hazard ratios are adjusted for type of military service and represent change in risk per quartile increase in cytokine concentration

The analyses of heterogeneity of cytokine associations by time interval from date of sample collection to date of cancer diagnosis, or age at cancer diagnosis (Tables 5 and 6, respectively), revealed several significant findings. The most consistent evidence of heterogeneity by time to diagnosis (22.7–7.2, 7.1–3.6, 3.5–1.9, 1.8–1.0 years) and age at diagnosis (<35, ≥35) was for IL-15 (*P* = 0.016 and *P* = 0.002, respectively). The inverse association with IL-15 was strongest >7.1 years prior to brain cancer diagnosis (HR = 0.75; 95% CI: 0.61, 0.92) and within 1.8–1.0 years of diagnosis (HR = 0.77, 95% CI: 0.63, 0.95), as well as in individuals ≥35 years old at cancer diagnosis (HR = 0.72; 95% CI: 0.58, 0.90).

**Table 5:** Associations between pre-diagnostic levels of serum cytokines and risk of adult brain cancer among active component military personnel according to quartiles of time between blood draw and diagnosis date

Cytokine		Time before diagnosis (years)				
		22.2–7.2	7.1–3.6	3.5–1.9	1.8–1.0	*P* ^a^
IL-12p40	HR^b^	1.26	1.05	0.98	1.03	0.101
	95% CI	1.04, 1.51	0.88, 1.25	0.82, 1.16	0.87, 1.23	
IL-15	HR	0.75	0.90	0.92	0.77	0.016
	95% CI	0.61, 0.92	0.72, 1.11	0.76, 1.11	0.63, 0.95	
IL-16	HR	0.90	0.84	0.82	0.87	0.787
	95% CI	0.75, 1.05	0.70, 1.01	0.68, 0.98	0.73, 1.03	
IL-7	HR	1.12	0.98	1.12	1.09	0.227
	95% CI	0.95, 1.33	0.82, 1.16	0.95, 1.32	0.93, 1.28	
MCP1	HR	0.95	0.92	1.05	0.87	0.123
	95% CI	0.78, 1.15	0.76, 1.10	0.88, 1.25	0.73, 1.04	
TARC	HR	0.98	0.92	1.08	1.12	0.165
	95% CI	0.82, 1.17	0.77, 1.10	0.92, 1.27	0.95, 1.33	
PLGF	HR	1.08	0.95	1.00	1.07	0.698
	95% CI	0.88, 1.31	0.77, 1.17	0.84, 1.20	0.88, 1.31	
VEGF	HR	1.03	1.16	0.99	1.06	0.828
	95% CI	0.85, 1.24	0.97, 1.40	0.85, 1.17	0.89, 1.25	
IFNg	HR	1.09	1.00	0.98	1.02	0.483
	95% CI	0.92, 1.29	0.83, 1.19	0.83, 1.17	0.87, 1.21	
IL-10	HR	0.87	0.92	0.92	0.87	0.297
	95% CI	0.71, 1.07	0.75, 1.12	0.75, 1.12	0.72, 1.05	
IL-8	HR	0.79	1.19	0.92	0.97	0.062
	95% CI	0.64, 0.97	0.97, 1.46	0.76, 1.13	0.78, 1.21	
TNFa	HR	0.99	1.20	0.96	1.00	0.219
	95% CI	0.81, 1.21	0.97, 1.47	0.79, 1.16	0.81, 1.24	
HGF	HR	0.86	0.98	1.09	0.90	0.015
	95% CI	0.71, 1.04	0.83, 1.17	0.92, 1.30	0.76, 1.05	
TGFb1	HR	1.04	1.03	1.19	1.22	0.403
	95% CI	0.86, 1.26	0.86, 1.25	0.97, 1.47	1.00, 1.49	

*HR* hazard ratio, *95% CI* 95% confidence interval

^a^*P* value for heterogeneity of quartile cytokine trends over time

^b^All hazard ratios are adjusted for type of military service and represent change in risk per quartile increase in cytokine concentration

**Table 6:** Associations between serum levels of pre-diagnostic cytokines and risk of adult brain cancer among active component military personnel according to age at cancer diagnosis

Cytokine		Age at diagnosis (years)		*P* ^a^
		≤35	>35	
IL-12p40	HR^b^	1.03	1.15	0.222
	95% CI	0.89, 1.20	0.94, 1.40	
IL-15	HR	0.89	0.72	0.002
	95% CI	0.77, 1.04	0.58, 0.90	
IL-16	HR	0.82	0.88	0.064
	95% CI	0.72, 0.93	0.75, 1.04	
IL-7	HR	1.11	1.02	0.831
	95% CI	0.98, 1.25	0.85, 1.22	
MCP1	HR	0.93	0.98	0.731
	95% CI	0.81, 1.06	0.81, 1.19	
TARC	HR	1.13	0.90	0.121
	95% CI	0.99, 1.30	0.74, 1.08	
PLGF	HR	1.02	0.99	0.986
	95% CI	0.88, 1.18	0.80, 1.22	
VEGF	HR	1.04	1.04	0.559
	95% CI	0.90, 1.21	0.85, 1.26	
IFNg	HR	1.02	1.06	0.895
	95% CI	0.90, 1.15	0.89, 1.27	
IL-10	HR	0.92	0.85	0.055
	95% CI	0.81, 1.04	0.70, 1.03	
IL-8	HR	0.97	0.96	0.212
	95% CI	0.85, 1.10	0.80, 1.14	
TNFa	HR	1.05	0.98	0.730
	95% CI	0.91, 1.21	0.83, 1.16	
HGF	HR	1.01	0.82	0.019
	95% CI	0.89, 1.14	0.68, 0.99	
TGFb1	HR	1.10	1.11	0.334
	95% CI	0.96, 1.25	0.91, 1.36	

*HR* hazard ratio, *95% CI* 95% confidence interval

^a^*P* value for heterogeneity of quartile cytokine trends by age

^b^All hazard ratios are adjusted for type of military service and represent change in risk per quartile increase in cytokine concentration

However, when evaluating effect modification of IL-15 by time or age and simultaneously allowing for modification by a history of diagnosis of immune-related condition, we found that the modifying effect of time and age was no longer significant (*P* = 0.753 and *P* = 0.172, respectively), while the modifying effect of immune-related condition with adjustment for time or age largely persisted (*P* = 0.024 and *P* = 0.057, respectively; data not shown).

## Discussion

In this nested case-control study of first primary brain cancer among active component military personal with serially collected pre-diagnostic serum samples, we found inverse, independent associations between levels of IL-15 and IL-16 and risk of cancer. We found no evidence that diagnosis of immune-related conditions ≥12 or ≥24 months prior to the date of cancer diagnosis was associated with risk of brain cancer overall; however, the brain cancer associations with IL-15 and IL-16 varied significantly by history of diagnosis of immune condition and were more pronounced in individuals with positive than negative history.

Our study provides new evidence concerning the possible importance of IL-15 and IL-16 in the aetiology of brain cancer with young and middle age onset. Our approach to studying the role of cytokines was to measure a limited number of candidates with strong prior evidence and well-performing assays in serially collected pre-diagnostic serum samples among active component military personnel. Due to the rarity of glioma, it is challenging to obtain sufficient numbers of pre-diagnostic samples for epidemiologic studies. Only one other study of glioma, conducted in Norway (JSBC study), assessed pre-diagnostic cytokine levels.^23,24^ While the current and JSBC study are similar in size (457 and 487 case/control sets, respectively), the JSBC investigators used a single pre-diagnostic serum sample to measure 277 cytokines.^23,24^ They found five cytokines (VEGF, beta-Catenin, CCL22, LIF, and sIL10RB) to be significantly associated with risk of glioma at different time periods prior to glioma diagnosis, i.e., discovery rate within the false positive range; the associations with IL-15 and IL-16 in that study were null.^24^

There are several possible reasons for the discrepancy of our findings and those of the JSBC study, including different methodological approaches and population characteristics. Our study was conducted among active component military personnel who are younger, fitter, and disproportionately male relative to the general population. As would be expected given the young age at diagnosis (median age, 30 years), 80% of cases with known histology in the current study (ACTUR cases) had low-grade astrocytoma, oligodendroglioma, or mixed oligoastrocytoma, while the remaining 20% had high-grade anaplastic oligodendroglioma or glioblastoma. By comparison, 65% of cases in the JSBC study (median age, 57 years) were glioblastoma.^23,24^ It is possible that the associations with different cytokines observed in the two studies reflect etiologic heterogeneity of histologically/genetically distinct glial tumours.^3,29^ However, alternative explanations include variable characteristics of assays used to measure cytokines, as well as chance.

Another important finding in our study is that IL-15 and IL-16 associations varied significantly by history of diagnosis of immune-related condition available from in-patient or out-patient medical records of military personnel. Based on results of previous epidemiologic studies^1–3,5,6^ including a hospital discharge records study of 4.5 million U.S. male veterans,^6^ we anticipated finding an inverse association between prior diagnosis of immune conditions and risk of brain cancer and thought that cytokine levels might be at least partially responsible for this association. Instead, we found no associations with immune-related conditions diagnosed either ≥12 months or ≥24 months prior; these results were unaffected by adjustment for cytokine levels. In addition to young age and preponderance of low grade neuroepithelial cancers discussed above, this might be due to a lower prevalence and lesser severity of allergies and autoimmune diseases in active duty military personnel related to medical selection and disqualification from service^30^ and thus low power. The lack of a main effect of immune-related conditions in our study does not preclude a role for these conditions in the aetiology of glioma insofar as a prior diagnosis of an immune-related condition significantly modified the associations of glioma risk with IL-15 and IL-16. Both cytokine associations were stronger in individuals with a positive than negative medical history, particularly for IL-15. The modifying effect of immune-related conditions on the IL-15 association persisted in analyses allowing for independent effect modification by lag-time interval before cancer diagnosis or age at cancer diagnosis, but neither effect modification by lag-time nor age remained significant. We do not know what factors associated with presence of allergy or autoimmune disease would contribute to a modifying effect, but unmeasured cytokines, genetic susceptibility, reactivity of immune cells, or chance could be involved.

An inverse relationship between pre-diagnostic levels of serum IL-15 and brain cancer risk is plausible biologically. One of the main effects of IL-15 is the induction of proliferation and differentiation of natural killer and CD8 memory cells and, thus, enhanced immune-surveillance against cancer.^31–33^ IL-15 in the brain originates either from the periphery, through limited penetration of the blood-brain-barrier, or from production within the brain by glial and neural cells; the IL-15 receptors are expressed throughout the brain.^32^ Intraperitoneal injection of ALT-803 (a complex of an IL-15 super-agonist and a dimeric IL-15 receptor) to glioblastoma-bearing mice was recently found to inhibit tumour growth, prolong survival, and eradicate tumour in 20% of surviving animals.^34^ Clinical trials of ALT-803 complex to treat patients with metastatic melanoma, kidney and bladder cancer have been promising;^33^ however, there have been no reports concerning glioma in humans thus far. Compared with IL-15, collective evidence for relevance of IL-16 to glioma aetiology is limited. IL-16 mainly functions as chemoattractant of immune cells expressing CD4 molecule. Higher levels of this cytokine were found in patients with multiple sclerosis, rheumatoid arthritis, and Crohn’s disease.^35^ Interestingly, allergic sensitivity to atopic dermatitis was positively correlated with serum levels of IL-16, as well as total IgE,^36,37^ although the correlation with IL-16 was weaker than the correlation with IgE levels. In addition, one study reported increased proportion of IL-16 expressing macrophages and microglial cells in human astrocytic tumours that correlated with tumour’s grade,^38^ while another study found increased risk of glioma with SNPs in *IL-16* gene.^39^

Our study has several strengths and limitations. It is nested in a cohort of active component military personnel, a well-defined population with standardised access to medical care and level of care. Another important strength of our study is that 67% of cases and controls had three serially collected pre-diagnostic serum samples allowing us to assess within subject temporal stability of cytokine concentration (as measured by ICCs) and lag-time variation in risk. Of 14 cytokines, half exhibited fair or adequate ICCs and another half exhibited poor ICCs. While the reasons for these differences are unclear, the main effect of low ICCs is reduced statistical power and thus an increased possibility of false negative findings. We used electronic records from two established medical registries (ACTUR and DMSS) to identify brain cancer cases and controls and to obtain in- and out-patient diagnoses of immune-related conditions (DMSS). We think that combined use of in- and out-patient records assured comprehensive ascertainment of serious immune-related conditions, although we cannot exclude a possibility of under-ascertainment of milder conditions, particularly those occurring prior to enrolment. Low prevalence of these conditions among military personnel discussed above and a relatively small number of informative case-control pairs among individuals with immune conditions might have limited our power to detect additional modifications of cytokine associations by immune status. Most cases identified through ACTUR had ICD-O-3 codes consistent with low grade astrocytoma, oligodendroglioma, or mixed oligoastrocytoma; however, only ICD-9 and CPT codes were available for cases identified through DMSS. We found no evidence that this may have introduced bias, as cytokine associations for DMSS and ACTUR cases were not meaningfully different. Nonetheless, the lack of detailed morphology codes for the DMSS cases restricted our ability to evaluate etiologic heterogeneity for histologically distinct primary brain cancers. To reduce assay-related variability, we plated all samples for a case-control set on the same plate in adjacent wells. The review of quality control indicators of cytokine assay performance confirmed that these performed well. We did not adjust for multiple testing because we assessed a limited number of cytokines that were selected based on prior hypotheses.

In conclusion, novel associations with pre-diagnostic serum levels of IL-15 and IL-16 and their modification by history of diagnosis of immune-related conditions support the importance of immune alterations in the aetiology of young- and middle-age-at-onset brain glioma years before diagnosis. Large studies of brain glioma including a broad range of histologic and molecular subtypes in independent populations are needed to validate and extend our findings.

### Disclaimer

The contents of this publication are the sole responsibility of the authors and do not necessarily reflect the views, assertions, opinions or policies of the Uniformed Services University of the Health Sciences (USUHS) or the Department of Defense (DoD).

## Electronic supplementary material

Supplemental Materials and Data

## Availability of data and materials

Data will be made available upon request.
