# Supplementary material for: Serially measured pre-diagnostic levels of serum cytokines and risk of brain cancer in active component military personnel
Source: Br J Cancer. 2018 Oct 9;119(7):893–900. doi: 10.1038/s41416-018-0272-x (PMC6189110; doi:10.1038/s41416-018-0272-x)
Supplement: Supplementary file 1 — Supplemental Materials and Data [file 41416_2018_272_MOESM1_ESM.docx]

**Supplemental Materials and Data**

**Table S1.** International Classification of Diseases 9th revision codes for pre-existing immune-related conditions extracted from the DMSS database in the study of adult brain cancer among active component military personnel

| **Allergy** | **ICD-9 code** |
| --- | --- |
| Hay fever | 477 |
| Asthma | 493 |
| Extrinsic allergic alveolitis | 495 |
| Atopic dermatitis and related conditions | 691 |
| Contact dermatitis and other eczema | 692 |
| Dermatitis due to food taken internally | 693.1 |
| Bullous dermatoses | 694 |
| Erythematous conditions | 695 |
| Urticaria | 708 |
| Allergic arthritis | 716.2 |
| Other anaphylactic shock not elsewhere classified elsewhereclcccclassified | 995.0 |
| Allergy unspecified | 995.3 |
| Allergy to other than medical agents | V15.0 |
| **Autoimmune disease** | **ICD-9 code** |
| ***Systemic*** |  |
| Rheumatoid arthritis | 714.0, 714.1, 714.2, 714.3, 714.81, V82.1 |
| Sjögren’s syndrome | 710.2 |
| Systemic lupus erythematosus | 710.0 |
| Systemic sclerosis    (scleroderma) | 710.1 |
| Sarcoidosis | 135 |
| Goodpasture's syndrome | 446.21 |
| Behçet's syndrome | 136.1 |
| ***Organ specific*** |  |
| Addison's disease | 255.4 |
| Amyotrophic lateral sclerosis | 335.20 |
| Ankylosing spondylitis | 720.0 |
| Aplastic anemia (idiopathic) | 284.9 |
| Autoimmune hemolytic anemia | 283.0 |
| Chronic rheumatic heart disease | 393-398 |
| Celiac disease | 579.0 |
| Crohn’s disease | 555 (555.0, 555.1, 555.2, 555.9) |
| Discoid lupus erythematosus | 695.4 |
| Dermatomyositis | 710.3 |
| Polymyositis | 710.4 |
| Graves' disease | 242.0 |
| Hashimoto's thyroiditis | 245.2 |
| Immune thrombocytopenic purpura | 287.31 |
| Diabetes mellitus type I  (controlled) | 250.×1. 250.×3 |
| Localized scleroderma | 701.0 |
| Meniere’s syndrome | 386.0, 386.00, 386.01, 386.02, 386.03,386.04 |
| Multiple sclerosis | 340 |
| Myasthenia gravis | 358.0, 358.00, 358.01 |
| Pernicious anemia | 281.0 |
| Polyarteritis nodosa | 446.0 |
| Vitiligo | 709.01, 374.53 |
| Systemic vasculitis(Wegener granulomatosis,  Churg-Strauss syndrome) | 446.4 |
| Polymyalgia rheumatica | 725 |
| Psoriasis | 696.0, 696.1 |
| Reiter's syndrome | 099.3 |
| Sarcoidosis | 135 |
| Ulcerative colitis | 556 (556.0, 556.1, 556.2, 556.3, 556.4, 556.5, 56.6, 556.8, 556.9) |
| Mooren’s ulcer | 370.07 |
| Vogt-Koyanagi-Harada disease | 364.24 |
| Alopecia areata | 704.01 |
| Pemphigus | 694.4 |
| Giant cell arteritis | 446.5 |
| Polyarteritis nodosa | 446.0 |
| Takayasu's disease | 446.7 |
| Chronic inflammatory demyelinating polyneuritis | 357.81 |

Abbreviations: DMSS = Defense Medical Surveillance System; ICD-9 **=** International Classification of Diseases 9th revision.

**Table S2.** Cytokine concentration cut-offs (pg/ml) used to define lower, middle, and upper quartiles in the analyses of cytokine trend

| **Cytokine** | **Q25** | **Q50** | **Q75** |
| --- | --- | --- | --- |
| IL-12p40 | 76.0 | 102.5 | 140.8 |
| IL-15 | 1.5 | 1.9 | 2.2 |
| IL-16 | 189.6 | 259.7 | 356.3 |
| IL-7 | 12.6 | 17.1 | 23.2 |
| MCP1 | 207.1 | 282.6 | 374.8 |
| TARC | 187.6 | 287.7 | 432.1 |
| PLGF | 19.9 | 23.9 | 28.8 |
| VEGF | 184.4 | 317.5 | 539.5 |
| IFNg | 3.3 | 4.6 | 6.8 |
| IL-10 | 0.33 | 0.39 | 0.74 |
| IL-8 | 14.0 | 43.0 | 183.3 |
| TNFa | 0.68 | 1.06 | 1.83 |
| HGF | 281.8 | 357.5 | 467.5 |
| TGFb1 | 25086.4 | 33140.9 | 41470.8 |

**Table S3.** Association between immune-related conditions diagnosed ≥24 months prior to the reference date and risk of brain cancer among active component military personnel

| **Immune-related condition** | **Controls**  **N=457 (%)** | **Cases**  **N=457 (%)** | **HR** | **95% CI** |
| --- | --- | --- | --- | --- |
| **Any allergy** | 99 | 93 | 0.86 | 0.60, 1.24 |
| Hay fever | 44 | 53 | 1.12 | 0.69, 1.84 |
| Asthma | 8 | 12 | 1.59 | 0.64, 3.96 |
| Eczema | 49 | 44 | 0.89 | 0.58, 1.38 |
| Other | 16 | 19 | 1.14 | 0.56, 2.32 |
| **Any autoimmune disease** | 14 | 16 | 1.08 | 0.50, 2.34 |
| Organ-specific | 8 | 14 | 1.99 | 0.81, 4.90 |
| Systemic | 2 | 0 | NE^a^ |  |
| **Any allergy or autoimmune disease** | 104 | 102 | 0.90 | 0.63, 1.29 |

Abbreviations: HR = Hazard ratio; 95% CI = 95% confidence interval; NE = not estimable.

^a^ P value for Fisher’s exact test = 0.12.

**Table S4.** Intraclass correlation coefficients (ICC) for cytokine concentration in controls with repeated serum samples

| **Cytokine** | **ICC (%)** |
| --- | --- |
| IL-12p40 | 68.6 |
| IL-15 | 62.3 |
| IL-16 | 15.3 |
| IL-7 | 43.6 |
| MCP1 | 39.7 |
| TARC | 56.2 |
| PLGF | 51.2 |
| VEGF | 57.1 |
| IFNg | 14.0 |
| IL-10 | 4.2 |
| IL-8 | 20.6 |
| TNFa | 20.2 |
| HGF | 41.5 |
| TGFb1 | 36.9 |

**Table S5.** Associations between pre-diagnostic levels of cytokines in serum samples collected ≥24 months prior to the reference date and risk of adult brain cancer among active component military personnel

|  |  |  |  |
| --- | --- | --- | --- |
| **Cytokine** |  | **Overall** | **P**^a^ |
| IL-12p40 | HR^b^ | 1.07 | 0.269 |
|  | 95% CI | 0.95, 1.21 |  |
| IL-15 | HR | 0.84 | 0.014 |
|  | 95% CI | 0.74, 0.97 |  |
| IL-16 | HR | 0.85 | 0.004 |
|  | 95% CI | 0.76, 0.95 |  |
| IL-7 | HR | 1.05 | 0.365 |
|  | 95% CI | 0.94, 1.18 |  |
| MCP1 | HR | 0.98 | 0.696 |
|  | 95% CI | 0.86, 1.11 |  |
| TARC | HR | 0.97 | 0.608 |
|  | 95% CI | 0.86, 1.09 |  |
| PLGF | HR | 1.02 | 0.797 |
|  | 95% CI | 0.89, 1.16 |  |
| VEGF | HR | 1.07 | 0.315 |
|  | 95% CI | 0.94, 1.21 |  |
| IFNg | HR | 1.03 | 0.641 |
|  | 95% CI | 0.92, 1.15 |  |
| IL-10 | HR | 0.91 | 0.133 |
|  | 95% CI | 0.80, 1.03 |  |
| IL-8 | HR | 0.98 | 0.679 |
|  | 95% CI | 0.87, 1.10 |  |
| TNFa | HR | 1.05 | 0.441 |
|  | 95% CI | 0.93, 1.18 |  |
| HGF | HR | 0.98 | 0.711 |
|  | 95% CI | 0.87, 1.10 |  |
| TGFb1 | HR | 1.09 | 0.185 |
|  | 95% CI | 0.96, 1.23 |  |

Abbreviations: HR = hazard ratio; 95% CI = 95% confidence interval.

^a^ P value for continuous quartile trend in cytokine concentration.

^b^All hazard ratios are adjusted for type of military service and represent change in risk per quartile increase in cytokine concentration.

**Table S6.** Associations between pre-diagnostic levels of serum cytokines and risk of adult brain cancer among active component military personnel according to source of case ascertainment: ACTRUR vs. DMSS

| **Cytokine** |  | **ACTUR** | **P^a^** | **DMSS** | **P^a^** |
| --- | --- | --- | --- | --- | --- |
| IL-12p40 | HR^b^ | 1.13 | 0.260 | 1.02 | 0.739 |
|  | 95% CI | 0.91, 1.41 |  | 0.89, 1.18 |  |
| IL-15 | HR | 0.83 | 0.077 | 0.80 | 0.004 |
|  | 95% CI | 0.67, 1.02 |  | 0.69, 0.93 |  |
| IL-16 | HR | 0.84 | 0.046 | 0.83 | 0.003 |
|  | 95% CI | 0.70, 1.00 |  | 0.73, 0.94 |  |
| IL-7 | HR | 1.03 | 0.772 | 1.09 | 0.190 |
|  | 95% CI | 0.85, 1.24 |  | 0.96, 1.23 |  |
| MCP1 | HR | 0.90 | 0.334 | 0.95 | 0.409 |
|  | 95% CI | 0.74, 1.11 |  | 0.83, 1.08 |  |
| TARC | HR | 1.07 | 0.491 | 0.99 | 0.914 |
|  | 95% CI | 0.88, 1.31 |  | 0.87, 1.14 |  |
| PLGF | HR | 0.94 | 0.547 | 1.01 | 0.888 |
|  | 95% CI | 0.75, 1.16 |  | 0.88, 1.17 |  |
| VEGF | HR | 0.95 | 0.624 | 1.09 | 0.218 |
|  | 95% CI | 0.77, 1.17 |  | 0.95, 1.26 |  |
| IFNg | HR | 1.20 | 0.038 | 0.94 | 0.338 |
|  | 95% CI | 1.01, 1.43 |  | 0.84, 1.06 |  |
| IL-10 | HR | 0.84 | 0.084 | 0.92 | 0.159 |
|  | 95% CI | 0.69, 1.02 |  | 0.81, 1.04 |  |
| IL-8 | HR | 0.88 | 0.147 | 1.03 | 0.660 |
|  | 95% CI | 0.74, 1.05 |  | 0.90, 1.17 |  |
| TNFa | HR | 1.04 | 0.696 | 1.01 | 0.845 |
|  | 95% CI | 0.86, 1.27 |  | 0.89, 1.15 |  |
| HGF | HR | 0.81 | 0.018 | 1.00 | 0.979 |
|  | 95% CI | 0.67, 0.96 |  | 0.88, 1.13 |  |
| TGFb1 | HR | 1.11 | 0.249 | 1.09 | 0.191 |
|  | 95% CI | 0.93, 1.34 |  | 0.96, 1.25 |  |

Abbreviations: ACTUR = Automated Central Tumor Registry; DMSS = Defense Medical Surveillance System; HR = hazard ratio; 95% CI = 95% confidence interval.

^a^ P value for continuous quartile trend in cytokine concentration.

^b^ All hazard ratios are adjusted for type of military service and represent change in risk per quartile increase in cytokine concentration.

**Table S7.** P value for a log-linear trend in cytokine concentration in individuals with and without immune-related condition

|  | **Immune-related condition** | |
| --- | --- | --- |
| **Cytokine** | No | Yes |
| IL-12p40 | 0.357 | 0.147 |
| IL-15 | 0.124 | 0.0003 |
| IL-16 | 0.065 | 0.046 |
| IL-7 | 0.634 | 0.224 |
| MCP1 | 0.821 | 0.116 |
| TARC | 0.795 | 0.833 |
| PLGF | 0.265 | 0.031 |
| VEGF | 0.093 | 0.606 |
| IFNg | 0.158 | 0.596 |
| IL-10 | 0.138 | 0.927 |
| IL-8 | 0.143 | 0.543 |
| TNFa | 0.912 | 0.939 |
| HGF | 0.900 | 0.844 |
| TGFb1 | 0.939 | 0.023 |
